# Subinhibitory Concentrations of Clinically-Relevant Antimicrobials Affect Resistance-Nodulation-Division Family Promoter Activity in *Acinetobacter baumannii*

**DOI:** 10.3389/fmicb.2021.780201

**Published:** 2021-12-03

**Authors:** Sonia Prieto Martin Gil, Ana Tajuelo, Mireia López-Siles, Michael J. McConnell

**Affiliations:** Intrahospital Infections Laboratory, National Center for Microbiology, Instituto de Salud Carlos III (ISCIII), Madrid, Spain

**Keywords:** *Acinetobacter baumannii*, efflux pumps, RND family, antibiotics, disinfectants, gene expression

## Abstract

Efflux pumps contribute to multidrug resistance in *Acinetobacter baumannii* due to their ability to expel a wide variety of structurally unrelated compounds. This study aimed to characterize the effect of subinhibitory concentrations of clinically-relevant antibiotics and disinfectants on the promoter activity of members of the Resistance-Nodulation-Division (RND) family in *A. baumannii*. The promoter regions from three RND efflux pumps (AdeABC, AdeFGH and AdeIJK) and the AdeRS regulatory system from three different *A. baumannii* strains (ATCC 17961, ATCC 17978, and ATCC 19606) were cloned into a luciferase reporter system (pLPV1Z). Promoter activity was quantitatively assessed in both exponential and stationary phase cultures after exposure to subinhibitory concentrations of four antibiotics from different classes (rifampicin, meropenem, tigecycline and colistin) and two disinfectants (ethanol and chlorhexidine). Subinhibitory concentrations of the compounds tested had variable effects on promoter activity that were highly dependent on the *A. baumannii* strain, the compound tested and the growth phase. Fold changes in AdeABC promoter activity ranged from 1.97 to 113.7, in AdeFGH from −5.6 to 1.13, in AdeIJK from −2.5 to 2, and in AdeRS from −36.2 to −1.32. Taken together, these results indicate that subinhibitory concentrations of clinically-relevant antibiotics and disinfectants affect the promoter activity of RND family members in *A. baumannii* in a strain and growth phase dependent manner. These results may have important implications for the treatment of infections caused by *A. baumannii*.

## Introduction

*Acinetobacter baumannii* is an opportunistic Gram-negative pathogen mainly associated with the healthcare environment, although incidence of infections caused by this microorganism in the community is increasing (Leung et al., 2006; Dijkshoorn et al., 2007; Lin and Lan, 2014). The most common and severe clinical manifestation of this pathogen is hospital-acquired pneumonia associated with mechanical ventilation, although it can cause various pathologies such as bacteremia, skin, soft tissue and urinary tract infections (Falagas and Rafailidis, 2007; McConnell et al., 2013). According to the 2017 WHO list of priority pathogens resistant to antibiotics (Taconnelli and Magrini, 2017), *A. baumannii* is considered within the critical group mainly due to the rapid appearance of multidrug resistant strains (Boucher et al., 2009). Particularly worrisome are descriptions of strains resistant to carbapenems, and to last-line therapeutic options such as colistin (Kyriakidis et al., 2021; Nang et al., 2021). The appearance of these extensively drug resistant strains, for which there are limited treatment options available, has increased the need for understanding the mechanisms underlying acquisition of resistance (Karakonstantis et al., 2020; Kyriakidis et al., 2021).

Although several mechanisms of antimicrobial resistance have been described for *A. baumannii* (Vila et al., 2007), efflux pumps are of special interest. Firstly, the primitive function of these secretion systems was the removal of metabolic end products or the expulsion of toxins or *quorum-sensing* molecules (Helling et al., 2002; Sánchez Díaz, 2003), suggesting that these transporters had a different biological function and were later adapted to the expulsion of antibiotics. This confers a non-specificity of substrate and may be related to their ability to actively export multiple, structurally-distinct classes of antimicrobials (Poole, 2002; Sánchez Díaz, 2003). Secondly, these efflux pumps are widely distributed as they have been identified in several microorganisms (Nikaido, 1994) and the presence of several genes coding for different pumps in bacterial genomes have been reported. They thus represent an important intrinsic resistance mechanism. Importantly, efflux pump overexpression has been associated with acquired resistance (Nikaido, 1994; Sánchez Díaz, 2003), which favors the emergence of mutants with a high degree of resistance (Sánchez Díaz, 2003).

Currently, six families of multidrug efflux systems have been associated with multidrug resistance in bacteria (Sánchez Díaz, 2003; Vila et al., 2007; Opazo et al., 2009), and at least five of them have been found in the genome of *A. baumannii* (Coyne et al., 2011). Among them, overexpression of the resistance-nodulation-division (RND) family, which includes pumps that are proton antiporters, has been associated with the development of multidrug-resistance in this species (Yoon et al., 2013; Salehi et al., 2021). All RND efflux pumps are a three-component based system composed of a transporter protein located in the cytoplasmic membrane, a membrane fusion protein in the periplasm and an outer membrane protein or porin (OMP) that is located in the outer membrane (Wieczorek et al., 2008). In *A. baumannii*, the RND family efflux pumps that have been identified include AdeABC, AdeIJK, AdeFGH, AdeDE and AdeXYZ. Whereas the former appears to be unique to *A. baumannii* species, AdeDE and AdeXYZ pumps have been found in other *Acinetobacter* species (Chu et al., 2006; Fournier et al., 2006). Regulatory systems have been described for some of these pumps such as the two-component AdeRS that is encoded upstream of *adeABC* (Marchand et al., 2004), AdeN a TetR transcriptional regulator which regulates AdeIJK (Rosenfeld et al., 2012) and AdeL, a LysR-Type regulator that overlaps with and regulates *adeFGH* (Coyne et al., 2010b).

Resistance-nodulation-division members have been associated with resistance to several antibiotics, but the environmental stimuli that lead to their activation/inhibition are not fully understood. Several studies have evaluated the contribution of efflux pumps in *A. baumannii* to resistance to a given compound by gene inactivation experiments or the use of efflux pump inhibitors (Magnet et al., 2001; Damier-Piolle et al., 2008; Coyne et al., 2010b; Rajamohan et al., 2010). However, few studies have characterized how pump expression varies as a consequence of exposure to subinhibitory concentrations of antimicrobials. In addition, while data exist with respect to several antibiotics, information regarding the effect of disinfectants is limited. Furthermore, most of these studies have focused on multidrug resistant strains (Ardebili et al., 2014; Lari et al., 2018) and the effect of antimicrobials on the expression of these pumps on non-resistant strains remains to be fully characterized, an aspect which could shed light on the emergence of resistance.

The aim of this study is to characterize how subinhibitory concentrations of antimicrobials affect the expression of members of the RND family in different *A. baumannii* strains. To achieve this, we studied the promoters that control the expression of the operons of three efflux pumps of the RND family (AdeABC, AdeFGH, and AdeIJK) and the two-component regulatory system (AdeRS) to explore changes in their expression. We employ a previously-validated plasmid reporter system that has been used for characterization of spatiotemporal gene expression dynamics in *A. baumannii* under multiple experimental conditions including exposure to UV light, chemical/disinfectant treatment, and iron depletion (Lucidi et al., 2019). This system was developed specifically for quantifying the activity of *Acinetobacter* promoters. We studied the effect of antibiotics with different mechanisms of action and disinfectants often used in the clinical setting, to cover the wide spectrum of substrates that can be released by this expulsion system.

## Materials and Methods

### Bacterial Strains and Culture Media

*Acinetobacter baumannii* ATCC^®^ 17978^TM^ (ATCC^®^ 17978), ATCC^®^19606^TM^ (ATCC^®^ 19606^*T)*^ and ATCC^®^ 17961^TM^ (ATCC^®^ 17961) were used for expression assays in order to cover different genetic backgrounds. *Escherichia coli* TOP10 was used as an intermediate host for all plasmid constructs. *E. coli* ATCC^®^ 25922^TM^, *Staphylococcus aureus* ATCC^®^ 29213^TM^ and *Pseudomonas aeruginosa* ATCC^®^ 27853^TM^ were used as control strains in minimum inhibitory concentration (MIC) experiments according to guidelines by Clinical and Laboratory–Standards Institute for antimicrobials (CLSI, 2017). All bacterial strains were grown routinely at 37°C in Luria-Bertani broth (LB) or on LB agar plates (Condalab) and supplemented with gentamicin at an appropriate concentration (10–100 μg/ml) for transformants selection. Mueller–Hinton broth II (MHB-II, Sigma) was used for growth in both MIC and luminescence assays. For long-term storage, bacterial cultures were grown on LB supplemented with 20% glycerol and stored at −80°C for further use.

### Susceptibility Testing

Minimum inhibitory concentration values were determined for a representative compound from different families of antimicrobials and disinfectants, covering a variety of mechanisms of action, and that included: colistin (COL), meropenem (MEM), rifampicin (RIF), tigecycline (TGC), chlorhexidine (CLX), and ethanol (EtOH). Product references and concentrations used are provided in Supplementary Table 1. Overnight cultures of each strain were adjusted to OD_600_ = 0.1 in MHB-II and the MIC was determined according to the recommendations of the Clinical and Laboratory Standards Institute for antimicrobials and the Sociedad Española de Enfermedades Infecciosas y Microbiología Clínica (Picazo, 2000; CLSI, 2017). For each strain, MIC values were determined at least by triplicate on three different days.

### Preparation of Electrocompetent Cells and Electroporation Conditions

Competent *E. coli* TOP10 cells were prepared for electrotransformation as follows. *E. coli* TOP10 cells were grown in LB at 37°C for 16 h. Cultures were refreshed by dilution 1:100 in 100 mL of LB and incubated for 3–4 h at 37°C with shaking at 200 rpm, until the OD_600_ reached ∼0.5. Cells were harvested by centrifugation (6,000 rpm for 10 min), washed four times with cold 10% glycerol, and suspended in 2 mL of 10% glycerol. One hundred-microliter aliquots of competent cells were stored at −80°C until further use.

*A. baumannii* electrocompetent cells were prepared as reported elsewhere (Lucidi et al., 2019) with minor modifications. Briefly, bacteria were grown in 10 mL of LB at 37°C for 16 h. Bacterial cultures were diluted 1:100 into 100 mL of LB and the cells were grown for 24 h at 37°C with shaking at 200 rpm. Cells were harvested and washed as indicated above. Eighty-microliter aliquots of competent cells were stored at −80°C until further use.

Electroporation was performed using one aliquot of competent cells and 100–250 ng of plasmid in electroporation cuvettes of 0.1 cm electrode gap (Bio-Rad). After pulsing with Bacteria default settings (1.8 kv, ≥ 3.9 mS), the cells were recovered in 1 mL of LB for 1 h at 37°C with shaking at 200 rpm and then transformants were selected on LB agar plates with the appropriate gentamicin concentration.

### Primer Design and Amplification of the Promoter Region

To carry out gene expression experiments, the operons corresponding to the *adeABC, adeFGH, adeIJK* efflux pumps and the *adeRS* control system were located in the genome of the *A. baumannii* ATCC 19606^*T*^ strain (Accession number: CP046654). The 500 bp upstream of the start codon of the first gene in the operon were selected. Primers (Table 1) were designed from the first and last 20 nucleotides of the 500 bp sequence, and the target sequences of *Bam*HI and *Not*I were included at the 5′ end of the forward and reverse primers, respectively. As the promoter regions of the four genes were highly similar among strains (≥98%, Supplementary Figure 1) one set of primers was used to amplify the 500 bp fragment in all the *A. baumannii* strains studied. All primers were obtained with desalted purification (Sigma).

**TABLE 1:** Primers used in this study and PCR conditions.

Target	Primer name	Sequence (5′–3′)^*a*^	Use	Tm^*b*^	Reference
*adeABC*	P_adeA_F	*ATTCG***GGATCC**CTTCATTTGGGTTAAAAGGCTTC	Promoter amplification	59.67	This study
	P_adeA_R	*ATTCG***GCGGCCGC**CATACTGTCCAAACCTAGTGAGTTT		57.02	This study
*adeFGH*	P_adeF_F	*ATTCG***GGATCC**CGATACAGGCACATCAATACGA	Promoter amplification	58.77	This study
	P_adeF_R	*ATTCG***GCGGCCGC**AGGTGCTCCTAGTTATTTGGATACC		59.85	This study
*adeIJK*	P_adeI_F	*ATTCG***GGATCC**TTGCACGCGTAGGCGG	Promoter amplification	60.02	This study
	P_adeI_R	*ATTCG***GCGGCCGC**GTTCCACCTCGTTTAGATAAAATCTA		58.33	This study
*adeRS*	P_adeR_F	*ATTCG***GGATCC**CTTTGAGTCTTGCTACCTCAGCTT	Promoter amplification	59.53	This study
	P_adeR_R	*ATTCG***GCGGCCGC**GATAATCTGGCTATAGAAAGTGCTTC		58.13	This study
pLPV1Z	T3 (modified)	GCAATTAACCCTCACTAAAGG	Constructs confirmation	54.09	Lucidi et al., 2019
	RBS	CTTAATTTCTCCTCTTTACTTACTT		51.46	This study

*^*a*^Boldface target of the *Bam*HI enzyme in forward (F) primers and of the *Not*I enzyme in reverse (R) primers, respectively.*

*Italics nucleotides indicate a random sequence included to facilitate the action of the enzyme.*

*^*b*^To calculate Tm, additional nucleotides to facilitate cleavage by restriction enzymes were not included.*

Genomic DNA was extracted with the freeze/thaw method and further used for promoter amplification through conventional PCR. The reaction mixture was composed of: 1 × EconoTaq PLUS 2X Master Mix (Sigma-Aldrich), 1 μM of forward primer, 1 μM of reverse primer and 1 μL of genomic DNA extract as template, in a total volume of 50 μL.

PCR conditions consisted of 10 min at 95°C for initial denaturation, followed by 30 cycles of 30 s at 95°C, 30 s at 58°C, and 30 s at 72°C and then a final extension of 10 min at 72°C. Products were visualized under UV light after gel electrophoresis on 1% (wt/vol) agarose gels in 1 × TAE buffer (Tris-acetate-EDTA, pH 8.0) stained with 1 × Gel Red 10,000 × (Biotium). The bands corresponding to the promoters were excised and the DNA was purified using the NucleoSpin Gel and PCR Clean-up kit (Macherey-Nagel) according to manufacturer recommendations.

### Plasmid Construction and Cloning Into *Acinetobacter baumannii* Strains

The pLPV1Z shuttle-vector, which includes the luciferase reporter systems (Lucidi et al., 2019), was used to evaluate changes in gene expression. A total of 12 plasmids were constructed including the promoters of the four operons, for the three *A. baumannii* strains of study. Both, the plasmid and the purified 500 bp amplicon including the promoters were double-digested with *Bam*HI and *Not*I (New England Biolabs) for 30 min and 37°C. After purification using the NucleoSpin Gel and PCR Clean-up kit (Macherey-Nagel) according to manufacturer recommendations, promoter and vector were ligated with T4 DNA ligase (New England Biolabs) at a plasmid:insert ratio of 1:20–1:50 at 16°C for 20 h. The final plasmid constructions were used to transform electrocompetent *E. coli* TOP10 cells. Transformants were selected by plating on LB agar supplemented with gentamicin (50 μg/ml) and screened by conventional PCR with T3 and RBS primers (Table 1), performed as described above.

Verified plasmid constructs were extracted and purified from a 10 mL *E. coli* TOP10 culture in LB supplemented with gentamicin 50 μg/mL using the NucleSpin Plasmid kit (Macherey-Nagel) following the protocol recommended by the manufacturer, and further confirmed by sequencing. Subsequently, constructs were introduced into electrocompetent cells of the corresponding strain of *A. baumannii* by electroporation. The clones incorporating the plasmid were selected by gentamicin resistance (10–100 μg/mL) according to the requirements of each strain (Table 2). Transformants were screened by conventional PCR with T3 and RBS primers as indicated previously, and also by measuring bioluminescence emission of the luciferase-luciferin system in the Orion II Microplate Luminometer (Berthold).

**TABLE 2:** Minimum inhibitory concentration (μg/mL) for *A. baumannii* strains ATCC 17978, ATCC 19606^*T*^ and ATCC 17961.

		*A. baumannii* strain		
Antimicrobial		ATCC 17978	ATCC 19606^*T*^	ATCC 17961
Disinfectant				
	Ethanol	6.25	3.13	3.13
	Chlorhexidine	16	16	16
Antibiotic				
	Gentamicin*	1	8	1.5
	Colistin	0.5	0.5	0.25
	Meropenem	0.25	1	0.5
	Rifampicin**	4	2	4
	Tigecycline	0.5	1	0.5

*MIC value determined by E-test strips.

**At least one of the control strains featured values not within the range established by the CLSI.

*The strains *E. coli* ATCC 25922, *S. aureus* ATCC 27853 and *P. aeruginosa* ATCC 29213 were used as controls and MIC values were within the expected range.*

### Qualitative Analysis of Promoter Activity

Agar diffusion assays were used to visually assess the effect of subinhibitory concentrations of antimicrobials on the expression of the operons. Briefly, cultures were prepared as described above, with a culture adjusted to OD_600_ = 0.1 to inoculate Mueller-Hinton agar plates (Oxoid) by swabbing. Up to 50 μL of the appropriate antimicrobial solution ranging from 25 × to 40,000 × of MIC (Supplementary Table 1) was placed in the center of the plate and incubated overnight at 37°C. A control with water was included as growth control and to assess the basal expression of the promoters in the absence of antimicrobials. Parental strains without any construct were also tested with all the antimicrobials as negative controls for bioluminescence. Bioluminescence was visualized using an IVIS Lumina XRMS series II *in vivo* Imaging System (Perkin Elmer) setting 1 s as exposure time for image capture.

### Quantitative Analysis of Promoter Activity

Initial experiments were carried out to optimize inoculum and antimicrobial concentration, as well as the most appropriate time for measurement (data not shown). The optimized protocol was used in the subsequent experiments to establish promoter activity. Briefly, *A. baumannii* strains carrying their respective construct of pLPV1Z:P*adeA*, pLPV1Z:P*adeF*, pLPV1Z:Pa*deR* and pLPV1Z:P*adeI* as well as the parental strain without plasmid were grown overnight at 37°C on LB agar with the appropriate gentamicin concentration. A subculture in LB maintaining the previous gentamicin concentration was incubated at 37°C for 20 h. These cultures were adjusted to an OD_600_ = 0.1 and then diluted 100-fold to inoculate 5 mL of MHB-II supplemented with the appropriate antimicrobial, at a concentration corresponding to 1/4 of the MIC (Supplementary Table 1). A growth control containing media without antimicrobial was included in all the experiments and was used for subsequent data normalization. To calculate background, a sample consisting of LB supplemented with antimicrobial was also included. The OD_600_ value and bioluminescence emission were recorded after incubation for 6 h (exponential phase) and 24 h (stationary phase) at 37°C and continuous shaking at 200 rpm. Cultures were grown in an incubator, and a 100 μl sample was taken at those time intervals to quantify OD_600_ on the one hand and bioluminescence on the other hand, using the Tecan Sunrise Absorbance Reader Analyser (Tecan) and an Orion II Microplate Luminometer (Berthold) reader, respectively. All experiments were performed at least in quadruplicate on four different days.

### Statistical Analysis

To account for differences in growth between strains and conditions, bioluminescence was normalized to the OD_600_ of the culture prior to fold-change calculation. To calculate the activity of the promoters, the normalized data was divided by that obtained from the corresponding growth control (culture without antimicrobial) in order to obtain the fold-change. For fold-change < 1 the inverse was calculated to obtain the real magnitude of the change and was expressed as negative growth in order to indicate decreased expression. In some cases, it was not possible to calculate the fold change of the strains because it did not grow sufficiently (OD_600_ < 0.02), and/or no growth was recorded after 24 h of incubation.

First, we determined if the fold-change calculated was different from 1 (as we established 1 as no changes in expression) by a one sample *T*-test. Secondly, the Kruskal–Wallis test was used to calculate differences in expression between strains and compounds. Further pairwise comparisons by subcategories of these variables were analyzed using the Dunn’s multiple comparison test. In this case, non-parametric statistical tests were used given the non-normal distribution of the data and the lack of homoscedasticity as assessed by Kolmogorov–Smirnov and Levene tests, respectively. Finally, correlation between expression was assessed by strain by calculating the Pearson correlation coefficient. In this case, the mean values of fold-changes of the four replicates performed in each antimicrobial treatment were used. Graphpad (Prism) and SPSS (IBM) were used for data plotting and statistical analysis. A *p*-value ≤ 0.05 was considered significant.

## Results

### Susceptibility to Antimicrobials

All three strains of *A. baumannii* were sensitive to the antimicrobials used according to CLSI and EUCAST breakpoint tables for interpretation of MIC values (CLSI, 2012; The European Committee on Antimicrobial Susceptibility Testing, 2021). However, each strain featured a different susceptibility profile (Table 2). *A. baumannii* ATCC 17961 had the lowest MIC for COL, whereas strain ATCC 17878 was the most sensitive to MEM. The *A. baumannii* ATCC 19606^*T*^ MIC of RIF was the lowest among the three strains tested. MIC values ranged between 0.25–16 μg/mL, and variation in the range of two- to fourfold were observed between strains. With regard to disinfectants, *A. baumannii* ATCC 17978 demonstrated a higher MIC for EtOH, which was twofold that of the other strains (Table 2). In contrast, the CLX MIC was similar for all the *A. baumannii* strains tested.

### Qualitative Analysis of Expression

**FIGURE 1:**
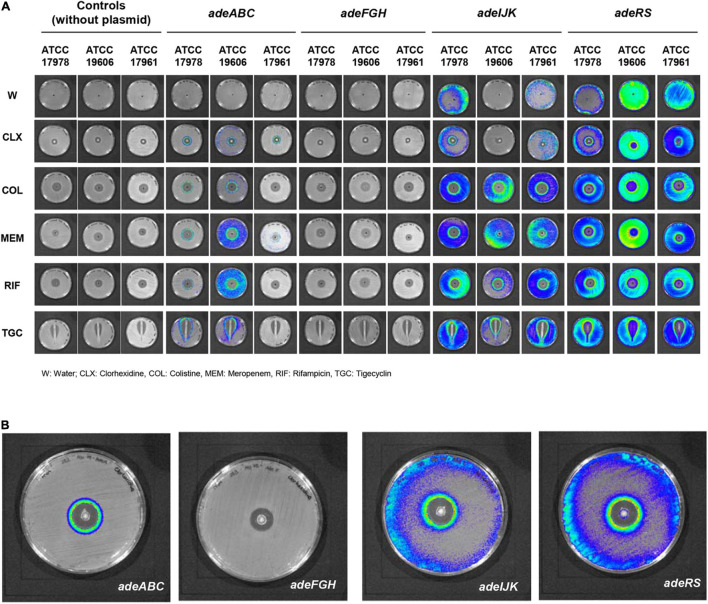
Qualitative analysis of promoter activity in the 12 plasmid constructions. **(A)** Bioluminescence emission of the different constructions when subjected to a concentration gradient of different compounds for all promoters. **(B)** Magnification of chlorhexidine treatments in strain ATCC17978 for *adeABC, adeFGH, adeIJK, adeRS* (from left to right). Luminescence intensity is represented by color from greatest to least as follows: red, yellow, green and blue.

We first aimed to characterize the bioluminescence produced by the different plasmid constructs when subjected to a concentration gradient of the different compounds to determine if there were qualitative differences in the expression from the RND promoters at subinhibitory concentrations of antimicrobials (Figures 1A,B).

In strains containing the *adeABC* promoter construct, a halo of bioluminescence was observed at subinhibitory concentrations of all the antimicrobials tested in strains ATCC 17978 and ATCC 19606^*T*^. In contrast, for strain ATCC 17961 the halo of bioluminescence was only observed in the presence of CLX and MEM.

In the case of the *adeFGH* promoter, all strains featured a rather similar pattern. No bioluminescence was observed for any of the antimicrobials.

With regard to expression of *adeIJK* promoter, assays with water revealed a low basal expression in the case of ATCC 17978 and ATCC 17961 strains but not in ATCC 19606^*T*^. A halo of increased bioluminescence was observed at subinhibitory concentrations in the presence of all the antimicrobials for ATCC 17978. This general phenomenon was also observed in the other two strains, but no bioluminescence was detected for strain ATCC 19606^*T*^ in the presence of CLX. For strain ATCC 17961, in assays with CLX only baseline levels of bioluminescence were observed. Altogether this result suggests a strain-specific response of this promoter to certain compounds.

Constitutive basal expression of *adeRS* promoters was also revealed in the absence of antimicrobials, especially for ATCC 19606^*T*^ and ATCC 17961. Nonetheless, halos of increased bioluminescence were observed with subinhibitory concentrations of all antimicrobials in all strains. This was especially clear in the presence of MEM in ATCC 19606^*T*^. These antibiotics also induced differences in expression in *adeABC*, suggesting that they may affect the expression of both the efflux pump and the regulatory system.

### Quantitative Analysis of Expression

To further study the effect of subinhibitory concentrations of antimicrobials on the expression of RND family members, quantitative assays were carried out by determining luciferase activity of the cultures treated with the different antimicrobials at 1/4 of the MIC. This allowed us to calculate fold changes as well as to determine whether there was overexpression or decreased expression of the promoter compared to growth in absence of antimicrobials.

**FIGURE 2:**
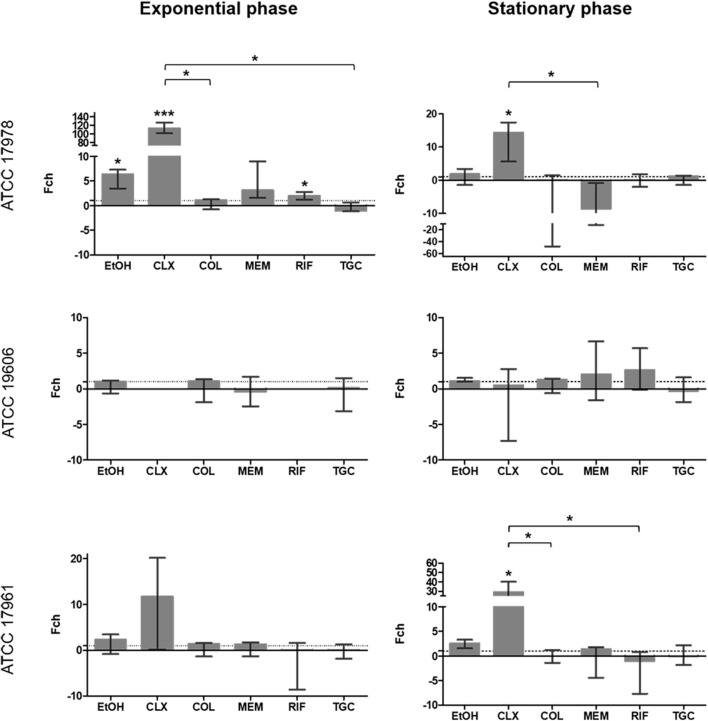
Fold change in *adeABC* promoter activity in the three strains included in the study calculated at 6 h (exponential phase) and 24 h (stationary phase) in the presence of subinhibitory concentrations of antibiotics. The fold-change represents the difference in luminescence. Values > 1 are considered as increased expression and values < 1 as decreased expression. Values = 1 represent no changes in expression. There is no representation of groups with no growth (OD_600_ < 0.02). Brackets indicate significant differences between treatments; *p < 0.05, and ***p < 0.001. Colistin (COL), meropenem (MEM), rifampicin (RIF), tigecycline (TGC), chlorhexidine (CLX), and ethanol (EtOH).

Significant overexpression of *adeABC* was observed for EtOH (*p* = 0.023), CLX (*p* = 0.0001) and RIF (*p* = 0.046) in strain ATCC 17978 (Figure 2). It is remarkable that CLX caused over a 100-fold-change at the exponential phase of growth. This effect was still significant, although smaller, during the stationary phase (*p* = 0.039). CLX also caused a 45-fold increase in expression of this promoter in strain ATCC 17961 during the stationary phase (*p* = 0.047). None of the compounds tested had a significant effect on the expression of the *adeABC* promoter in strain ATCC 19606 during exponential and stationary growth.

**FIGURE 3:**
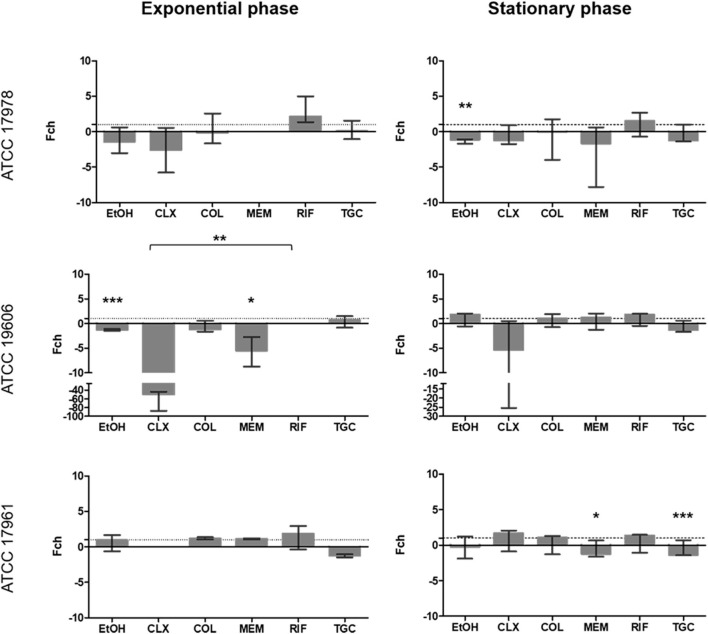
Fold change in *adeFGH* promoter activity in the three strains included in the study calculated at 6 h (exponential phase) and 24 h (stationary phase) in the presence of subinhibitory concentrations of antibiotics. The Fold-change represents the difference in luminescence. Values > 1 are considered as increased expression and values < 1 as decreased expression. Values = 1 represent no changes in gene expression. There is no representation of groups with no growth (OD_600_ < 0.02). Brackets indicate significant differences between treatments; *p < 0.05, **p < 0.01 and ***p < 0.001. Colistin (COL), meropenem (MEM), rifampicin (RIF), tigecycline (TGC), chlorhexidine (CLX), and ethanol (EtOH).

For *adeFGH* promoter expression, almost all fold-change values were around 1 or below (Figure 3). This suggested no overexpression from these promoters at subinhibitory concentrations of the antimicrobials and was in line with the results observed in the qualitative assay showing no bioluminescence. Only EtOH (*p* = 0.001) showed significantly decreased expression during the stationary phase in strain ATCC 17978. For strains ATCC 19606^*T*^ and ATCC 17961, significant effect of the antimicrobials was observed only during exponential phase. EtOH (*p* = 0.0001) and MEM (*p* = 0.026) significantly decreased the expression of *adeFGH* in strain ATCC 19606^*T*^. For strain ATCC 17961, MEM and of TGC had a significant effect on the expression from this promoter, however, they had opposite effects on promoter activity. Whereas MEM caused a 1.125-fold overexpression (*p* = 0.034), TGC caused a 1.25-fold decrease in expression (*p* = 0.0001).

**FIGURE 4:**
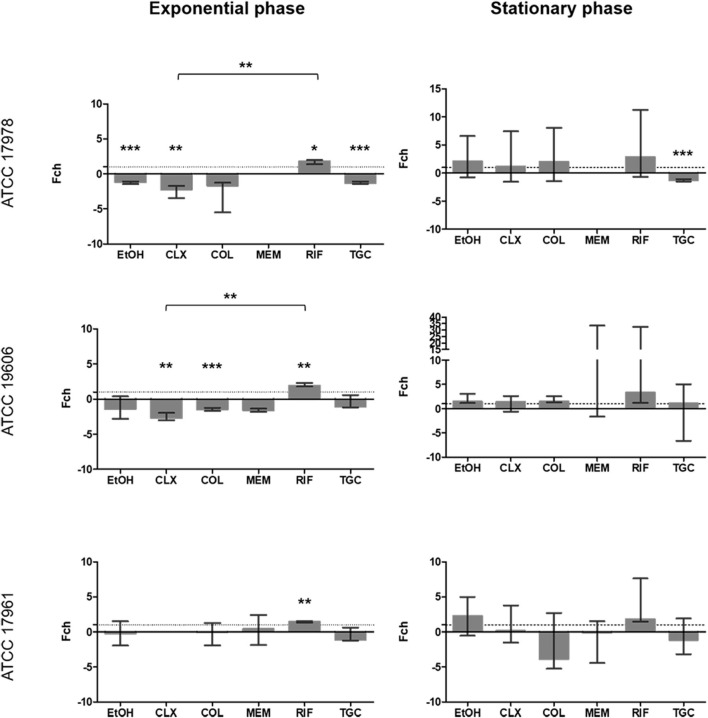
Fold change in *adeIJK* promoter activity in the three strains included in the study calculated at 6 h (exponential phase) and 24 h (stationary phase) in the presence of subinhibitory concentrations of antibiotics. The Fold-change represents the difference in luminescence. Values > 1 are considered as increased expression and values < 1 as decreased expression. Values = 1 represent no changes in gene expression. There is no representation of groups with no growth (OD_600_ < 0.02). Brackets represent significant differences between treatments; *p < 0.05, **p < 0.01 and ***p < 0.001. Colistin (COL), meropenem (MEM), rifampicin (RIF), tigecycline (TGC), chlorhexidine (CLX), and ethanol (EtOH).

With regards to expression from the *adeIJK* promoter (Figure 4), the effect on expression was mainly observed during the exponential phase. In strain ATCC 19606^*T*^, CLX, COL and MEM significantly decreased the expression of this promoter (*p* ≤ 0.0001). For strain ATCC 17978, EtOH (*p* = 0.0001), CLX (*p* = 0.006) and TGC (*p* = 0.0001) also showed a significant downregulation. Interestingly, RIF caused a significant overexpression of this pump in all strains (*p* ≤ 0.025), ranging between 1.44 to 2-fold increase in expression. In the stationary phase, only a significant decreased expression was observed for TGC in strain ATCC 17978 (*p* = 0.0001).

**FIGURE 5:**
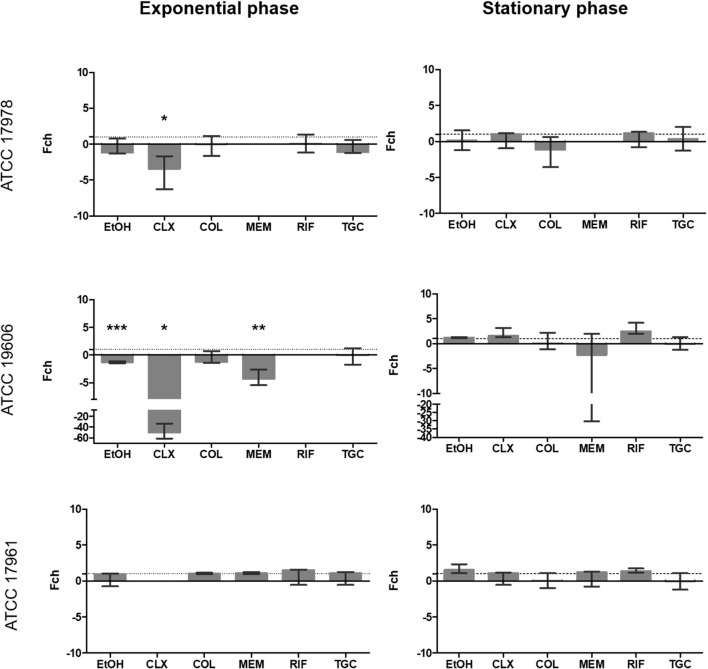
Fold change in *adeRS* promoter activity in the three strains included in the study calculated at 6 h (exponential phase) and 24 h (stationary phase) in the presence of subinhibitory concentrations of antibiotics. The Fold-change represents the difference in luminescence. Values > 1 are considered as increased expression and values < 1 as decreased expression. Values = 1 represent no changes in gene expression. There is no representation of groups with no growth (OD_600_ < 0.02); *p < 0.05, **p < 0.01 and ***p < 0.001. Colistin (COL), meropenem (MEM), rifampicin (RIF), tigecycline (TGC), chlorhexidine (CLX), and ethanol (EtOH).

For the *adeRS* promoter (Figure 5), EtOH, CLX, and MEM significantly decreased the expression from this promoter (*p* ≤ 0.026) during the exponential phase for strain ATCC 19606^*T*^. The same effect was observed for CLX in strain ATCC 17978 (*p* = 0.032). No significant effect on the stationary phase was observed for any of the antimicrobials in the other two strains.

It is important to point out that the apparent inconsistencies between qualitative (Figure 1) and quantitative assays (Figures 2–5) are due to technical limitations of the qualitative assay, related to the detection limit. This assay represents an end-point picture where overexpression of promoters can be observed through increases in luminescent intensity. In these images, no changes in expression or reduced expression would be observed as no bioluminescence. In contrast, in the quantitative assay, by comparison with the control without antimicrobial, a decrease in expression can also be calculated. This is the case of *adeFGH*, for example, where quantitative analysis demonstrated that almost all fold-change values were around 1 or below, and therefore not detected in the qualitative assay.

### Strain-Specificity of Promoter Activity

**FIGURE 6:**
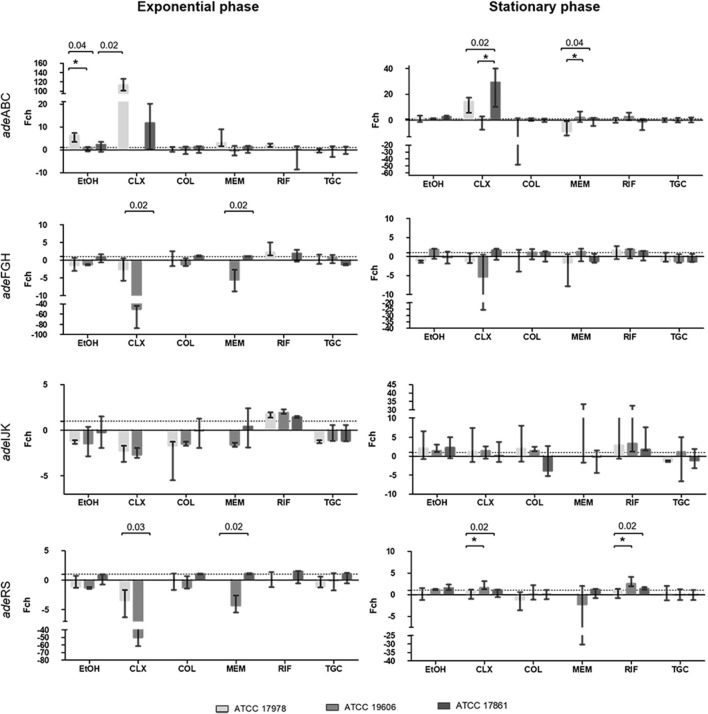
Comparison between strains with respect to fold-change obtained for the same compound and promoter. There is no representation of groups with no growth (OD_600_ < 0.02). The *p*-value obtained for a treatment is indicated by its value when it is significant and below it is represented with *p < 0.05, **p < 0.01 and **p < 0.001 between which strains there is a significant difference. Colistin (COL), meropenem (MEM), rifampicin (RIF), tigecycline (TGC), chlorhexidine (CLX), and ethanol (EtOH).

Data from the quantitative expression assays were used to assess if the tested compounds affected the expression of the four promoters similarly in the three *A. baumannii* strains used in this study. Altogether, the result of this analysis showed differences among strains in promotors expression under exposure to certain compounds and also depending on growth phase of the culture (Figure 6).

For the *adeABC* promoter, significant differences between strains were observed in four out of the six antimicrobials tested. During the exponential growth phase, differences in the effect of EtOH and CLX were observed (*p* ≤ 0.03). In the stationary phase, significant differences were also obtained for CLX (*p* = 0.015) and MEM (*p* = 0.04). Two antimicrobials affected *adeFGH* promoter differently between strains. In this case, during exponential phase significant differences were found for CLX and MEM (*p* = 0.02). For the *adeIJK* promoter, no statistically significant differences were observed between strains with any of the compounds tested, regardless of growth phase. For the *adeRS* promoter, significant differences were observed with CLX and MEM treatments (*p* ≤ 0.03) in the exponential phase. In the stationary phase there were also significant differences after treatment with CLX and RIF (*p* ≤ 0.024). Overall, CLX was the compound that featured more differences in expression among strains, regardless of the growth phase.

### Correlation Between Promoter Expression and Strain

To estimate if the expression of the different RND family members were related, based on the strain tested, for each strain we calculated pairwise correlations of the mean fold-changes obtained with all the antimicrobials tested together (Table 3). In general, few significant correlations were observed. During the exponential phase, negative correlation between *adeABC* and *adeRS* was found in strain ATCC 17978 whereas positive correlation between *adeFGH* and *adeRS* was found in strain ATCC 19606^*T*^. In the stationary phase, positive correlation between *adeIJK* with *adeABC* and *adeRS* were found in strains ATCC 19606^*T*^ and ATCC 17961, respectively.

**TABLE 3:** Pearson correlation of promoter activity fold-changes by strain, considering all antimicrobial treatments, in exponential and stationary phases.

		*A. baumannii* strain					

		ATCC 17978		ATCC 19606^*T*^		ATCC 17961	
		Corr. cof	*p*-value	Corr. cof	*p*-value	Corr. Cof	*p*-value
**Exponential phase**							
	*adeABC-adeFGH*	−0.696	0.192	0.223	0.777	−0.152	0.807
	*adeABC-adeIJK*	−0.384	0.523	−0.399	0.601	−0.765	0.131
	*adeABC-adeRS*	−**0.986**	**0.002**	0.245	0.755	−0.221	0.720
	*adeFGH-adeIJK*	0.773	0.125	0.858	0.063	0.667	0.219
	*adeFGH-adeRS*	0.781	0.119	**1.000**	**<0.001**	0.540	0.348
	*adeIJK-adeRS*	0.467	0.428	0.851	0.068	0.321	0.598
**Stationary phase**							
	*adeABC-adeFGH*	0.236	0.652	0.789	0.062	0.577	0.231
	*adeABC-adeIJK*	−0.111	0.859	**0.858**	**0.029**	0.050	0.924
	*adeABC-adeRS*	0.844	0.072	−0.376	0.463	−0.084	0.875
	*adeFGH-adeIJK*	0.429	0.471	0.387	0.448	0.345	0.503
	*adeFGH-adeRS*	0.353	0.560	−0.251	0.631	0.153	0.772
	*adeIJK-adeRS*	−0.113	0.856	−0.386	0.450	**0.884**	**0.019**

*Significant *p*-values (*p* ≤ 0.05) are highlighted in bold.*

## Discussion

Efflux pumps contribute to multidrug resistance in *A. baumannii* due to their ability to expel a wide variety of structurally unrelated compounds. In this study we analyzed the effect of subinhibitory concentrations of antimicrobials on the expression of four members of the RND family in different strains of *A. baumannii*. This approach allowed to screen a large number of combinations of strains, antimicrobials and promoter regions.

Our qualitative data demonstrated basal levels of expression of *adeRS* and *adeIJK* whereas expression of *adeABC* and *adeFGH* seems to be inducible. Constitutive expression of *adeIJK* has been previously reported (Damier-Piolle et al., 2008; Leus et al., 2018) whereas this is not the case for *adeFGH* (Coyne et al., 2010b, 2011) and overexpression of *adeABC* has been reported as a mechanism of acquired resistance (Magnet et al., 2001). Regardless of their basal expression, our study demonstrates that subinhibitory concentrations of clinically-relevant antimicrobials affect the promoter activity of all the members of the RND family included in this study. However, we have observed that none of the antimicrobials universally affects all pumps, or the regulatory system.

Our data show that EtOH, CLX, and RIF affected *adeABC* promoter expression. This is in agreement with previous studies (Camarena et al., 2010; Rajamohan et al., 2010; Hassan et al., 2013; Fernández-Cuenca et al., 2015; Lin et al., 2017) mainly using the ATCC 17978 strain (Camarena et al., 2010; Hassan et al., 2013) or clinical isolates (Lin et al., 2017). Whereas previous studies report similar data for disinfectants (Camarena et al., 2010; Hassan et al., 2013), our study is the first to demonstrate that subinhibitory concentrations of RIF affect promoter activity. Notably, we observed that subinhibitory concentration of CLX can induce up to a 130-fold increase in promoter activity of this efflux pump in a particular strain. This is in line with several studies reporting increased expression of this efflux pump when exposed to CLX (Rajamohan et al., 2010; Hassan et al., 2013; Fernández-Cuenca et al., 2015; Lin et al., 2017), including subinhibitory concentrations (Hassan et al., 2013; Lin et al., 2017), and supports previous reports indicating that among the tree efflux pumps included in this work, AdeABC appears to be the most affected by this compound (Rajamohan et al., 2010).

With regards to *adeFGH*, we observed a significantly decreased expression with EtOH, MEM and TGC. On the one hand, this could be due to the fact that AdeFGH is not constitutively expressed and therefore it would not contribute to intrinsic resistance (Coyne et al., 2010a,b). On the other hand, it could be the consequence or a result of other metabolic processes taking place inside the bacterial cell. For instance, EtOH concentrations similar to that used in the present study have been shown to enhance *A. baumannii* ATCC 17978 growth, and therefore AdeFGH repression may respond to a mechanism to maintain this compound within the cell (Smith et al., 2004). In the case of MEM, downregulation can respond to the presence of other resistance mechanisms that are more efficient, such as carbapenemases (Lee et al., 2010), which is in agreement with studies on other strains of *A. baumannii* showing that β-lactams are not a substrate of this pump (Coyne et al., 2010b, 2011). Conflicting data is reported in the literature concerning the effect of TGC on the *adeFGH* promoter. Whereas an overexpression of this pump has been associated with reduced susceptibility to TGC (Coyne et al., 2010b), our results revealed downregulation of this promoter when exposed to subinhibitory concentrations of this antibiotic, which is in agreement with a study reported by Navidifar et al. (2019) assessing exposure to concentrations within the MIC range of different strains.

Moderately increased activity of the *adeIJK* promoter was observed at subinhibitory concentrations of RIF in all three stains tested, which is consistent with the fact that this antibiotic is a substrate of this pump (Damier-Piolle et al., 2008). In contrast, *adeIJ*K expression was significantly reduced by EtOH, CLX, COL, MEM, and TGC. This could be the consequence of redundant metabolic processes within the cell, for instance AdeIJK has been reported to act synergistically with AdeABC to expel TGC (Damier-Piolle et al., 2008). However, it has also been reported that AdeIJK overexpression could be toxic (Damier-Piolle et al., 2008), and therefore its expression may be strictly regulated, in order to maintain non-toxic levels (Coyne et al., 2011). Taken together, our data support previous studies suggesting that this pump may contribute only to intrinsic resistance (Chu et al., 2006; Damier-Piolle et al., 2008; Coyne et al., 2010a). Nonetheless, in our approach the chromosomal copy of *adeIJK* has not been removed, and therefore, we cannot rule out that by increasing the number of copies of this promoter, the regulator mechanisms of this pump, the TetR transcriptional regulator AdeN (Rosenfeld et al., 2012), had been activated. Also, the different methodologies could explain the conflicting data that exists for some compounds such as MEM or CLX, ranging from no effect on *adeIJK* expression (Navidifar et al., 2019) to overexpression of this gene (Rajamohan et al., 2010; Leus et al., 2018).

We observed decreased expression of *adeRS* with subinhibitory concentrations of EtOH, MEM and CLX only during the exponential phase. In this two-component regulatory system, AdeR is the transcriptional regulator and AdeS is a sensor protein kinase, which detects changes in environmental conditions and activates AdeR, which ultimately results in changes in the expression of the AdeABC efflux pump (Magnet et al., 2001; Marchand et al., 2004). In this case, downregulation of *adeRS* can lead to overexpression of *adeABC* and thus result in antimicrobial resistance. Whereas we observed that the two disinfectants affected *adeRS* expression in several strains, the antibiotic MEM affected its expression exclusively in the ATCC 19606^*T*^ strain. To the best of our knowledge, no study has assessed the effect of antimicrobials on the expression of this regulatory system. Nonetheless, point mutations leading to amino acid substitutions in AdeRS have been linked to AdeABC overexpression and carbapenem resistance (Zhang et al., 2017). Despite the fact that the 500 bp upstream of the genes analyzed in this study are very similar between strains, it is possible that the binding sequences of other local or global regulators that naturally affect the expression of *adeRS* (Wieczorek et al., 2008) have been lost, and that therefore we are not detecting all changes in promoter activity. Further studies to analyze the effect of antimicrobials subinhibitory concentrations in AdeN and AdeL which regulate *adeFGH* and *adeIJK* expression, respectively, would be of interest.

Another aspect that our study evidenced is that antimicrobials affect promoter activity in a strain dependent manner. Differences between strains in the expression from *adeABC, adeFHG* and *adeRS* promoters were observed at subinhibitory concentrations of EtOH, CLX, MEM and RIF. Strain-dependent responses upon exposure to EtOH, MEM and CLX have been previously reported by Lin et al. (2017). While the former evaluates several genes including *adeB*, *adeJ* and *adeG* and observes this discordance, the latter describes how the control that *adeRS* exerts on *adeABC* is strain-specific. Thus, this phenomenon appears to occur with the regulatory system, and according to our data may affect all the pumps studied.

Nonetheless, within the same strain some compounds affected the expression of several promoters. For instance, CLX significantly affected the expression of *adeABC*, *adeIJK* and *adeRS* in strain ATCC 17978, and we corroborated negative correlation between the former and the latter, which agrees with the fact that AdeRS is a regulatory system of AdeABC. In strain ATCC 19606^*T*^ EtOH and MEM significantly decreased the expression of AdeFGH and AdeRS, and thus positive correlation was found between them in the exponential phase. This may indicate that regulation of RND family members is interconnected. Although few studies have explored the potential association between RND family members, some reports have shown this possibility. For instance inactivation of *adeFGH* has been reported to trigger overproduction of AdeABC (Leus et al., 2018), and AdeABC and AdeIJK have been shown to contribute synergistically to TGC resistance (Damier-Piolle et al., 2008). Without considering the strain and effect (downregulation or overexpression), we observed that EtOH affected the expression of all the promoters analyzed, and CLX and MEM at least three out of the four RND members studied, including the regulatory system and two efflux pumps. In line with this, it was previously reported that the same compound can be exported by several pumps, since efflux pumps represent an important mechanism of resistance as they can expel different compounds with varied structures (Sánchez Díaz, 2003).

Lastly, we observed that even within a strain, a similar pump and compound, differences of the effect were also observed depending on the growth phase. The only compounds that affected *adeABC*, *adeFGH* and *adeIJK* promoters regardless of growth phase were CLX, EtOH and TGC, respectively. In general, most of antimicrobials affected promoter expression during the exponential phase, and fold-changes were higher than those observed in the stationary phase. This may be due to the fact that, during the exponential phase, mechanisms to expel metabolism byproducts are more active. To the best of our knowledge, previous studies analyzing the effects of antimicrobials on RND members have focused either on the exponential phase (Camarena et al., 2010; Coyne et al., 2010a,b; Hassan et al., 2013; Lin et al., 2017) or stationary phase (Navidifar et al., 2019) but none compared both. Our study complements previous reports indicating that the effect of subinhibitory concentrations on promoter expression was growth phase dependent in some cases, which may have relevance for antimicrobial therapy, for example, in the context of biofilms.

## Conclusion

Altogether, the results obtained in this work support the idea that subinhibitory concentrations of antimicrobials have an effect on the expression of both efflux pumps and the regulatory systems of the RND family in *A. baumannii*. Future work aimed at defining the effect of antimicrobials on other regulatory elements involved in efflux pump regulation (e.g., AdeN), and the effect of additional clinically relevant antibiotics (e.g., third generation cephalosporins) using this system may be of interest for obtaining a more complete picture of efflux pump regulation. Furthermore, the fact that not all antibiotics produce a significant effect on all pumps is evident. Moreover, the effect they produce was shown to be highly dependent upon the strain, promoter and growth phase, suggesting that the gene content of the tested strain influences promotor activity. Since the effect of the compounds is not universal, it seems that the acquisition of resistance depends on a combination of several mechanisms, which requires further study.

## Data Availability Statement

The raw data supporting the conclusions of this article will be made available by the authors upon request, without undue reservation.

## Author Contributions

SPMG performed antimicrobials susceptibility testing, plasmid construction, quantitative analysis of expression, analyzed data, drafted the manuscript, and revised the final version. AT performed qualitative analysis of expression and revised the final version of the manuscript. ML-S assisted in plasmid construction, quantitative and qualitative analysis of expression, data analysis, supervised the study and drafted the manuscript. MJM conceived and supervised the study, revised the manuscript and granted funding. All authors contributed to the article and approved the submitted version.

## Conflict of Interest

MJM is founder and stockholder of the biotechnology spin-off company Vaxdyn, which develops vaccines for infections caused by MDR bacteria. Vaxdyn had no role in the elaboration of this manuscript. The remaining authors declare that the research was conducted in the absence of any commercial or financial relationships that could be construed as a potential conflict of interest.

## Publisher’s Note

All claims expressed in this article are solely those of the authors and do not necessarily represent those of their affiliated organizations, or those of the publisher, the editors and the reviewers. Any product that may be evaluated in this article, or claim that may be made by its manufacturer, is not guaranteed or endorsed by the publisher.
