# Supplementary material for: Subinhibitory Concentrations of Clinically-Relevant Antimicrobials Affect Resistance-Nodulation-Division Family Promoter Activity in Acinetobacter baumannii
Source: Front Microbiol. 2021 Dec 3;12:780201. doi: 10.3389/fmicb.2021.780201 (PMC8678518; doi:10.3389/fmicb.2021.780201)
Supplement: Supplementary file 1 [file Data_Sheet_1.docx]

Supplementary Material

**Supplementary Table1**. Antimicrobials analysed in this study and concentrations used in each assay.

|  |  |  | **MIC** | | **Quantitative expression assay** | | | | | **Qualitative expression assay** |
| --- | --- | --- | --- | --- | --- | --- | --- | --- | --- | --- |
| **Antimicrobial** | **Reference** | **Family** | **Concentration of stock solution** | **First concentration used in assays** | ***A. baumannii* ATCC 17978** | | ***A. baumannii* ATCC 19606^T^** | | ***A. baumannii* ATCC 17961** | **Concentration of stock solution in wells** |
| Gentamicin | Biomerieux, 412368 | Antibiotic, Aminoglycoside | 256-0.016µg/ml | * | * | * | | * | | * |
| Colistin | Panreac, A2922,0001 | Antibiotic, Polypeptide | 10mg/ml | 32 µg/ml | 0.125 µg/ml | 0.125 µg/ml | | 0.062 µg/ml | | 10mg/ml |
| Meropenem | Sigma, M2574-10MG | Antibiotic, Carbapenemic | 5mg/ml | 4 µg/ml | 0.625 µg/ml | 0.25 µg/ml | | 0.625 µg/ml | | 5mg/ml |
| Rifampicin | Sigma, R3501-250MG | Antibiotic, Macrocyclic (rifamycin) | 10mg/ml | 64 µg/ml | 1 µg/ml | 0.5 µg/ml | | 1 µg/ml | | 5mg/ml |
| Tigecycline | Sigma, Ref: PZ0021-5MG | Antibiotic, Glicilcicline (tetracycline) | 1 mg/ml | 4 µg/ml | 0.125 µg/ml | 0.25 µg/ml | | 0.125 µg/ml | | 1 mg/ml |
| Clorhexidine | Sigma, 282227-1G | Disinfectant, Chlorophenylbiguanide derivative | 0.4mg/ml | 32 µg/ml | 4 µg/ml | 4 µg/ml | | 4 µg/ml | | 2 mg/ml |
| Ethanol | Panreac, 141086.1212 | Disinfectant, Alcohol | 96% | 48% | 1.56 µg/ml | 0.78 µg/ml | | 0.78 µg/ml | | ** |

* Used for selection of clones incorporating the plasmid

** Not tested due to volatility

***10 20 30 40 50 60 70 80 90 100***

***....|....|....|....|....|....|....|....|....|....|....|....|....|....|....|....|....|....|....|....|***

**AdeA17978** CTTCATTTGGGTTAAAAGGCTTCACCACAAAGTCATCTGCACCTATGCGTAATGCCATAACTTTATCAATATCTTGATCTAACGCCGTCAACATGATCAC

**AdeA19606** CTTCATTTGGGTTAAAAGGCTTCACCACAAAGTCATCTGCACCTATGCGTAATGCCATAACTTTATCAATATCTTGATCTAGCGCCGTCAGCATGATCAC

**AdeA17961** CTTCATTTGGGTTAAAAGGCTTCACCACAAAGTCATCTGCACCTATGCGTAATGCCATAACTTTATCAATATCTTGATCTAGCGCCGTCAGCATGATCAC

***110 120 130 140 150 160 170 180 190 200***

***....|....|....|....|....|....|....|....|....|....|....|....|....|....|....|....|....|....|....|....|***

**AdeA17978** GGGAGTCTGAGCTTTTTGGCGTATTTTATTTAACACTTCCCAACCGTTTAATTCGGGTAATTTAATATCAAGTAAGATTAAATCGATGGGTTGGCTCGCA

**AdeA19606** GGGAGTCTGAGCTTTTTGGCGTATTTTATTTAATACTTCCCAACCGTTTAATTCGGGTAATTTAATATCAAGTAAGATTAAATCGATGGGTTGGCTAGCG

**AdeA17961** GGGAGTCTGAGCTTTTTGGCGTATTTTATTTAATACTTCCCAACCGTTTAATTCGGGTAATTTAATATCAAGTAAGATTAAATCGATGGGTTGGCTAGCG

***210 220 230 240 250 260 270 280 290 300***

***....|....|....|....|....|....|....|....|....|....|....|....|....|....|....|....|....|....|....|....|***

**AdeA17978** TGCAATTCAATTGCTTGCTTTCCATTCATGGCCCGAATAACACTCATGCCTTCACGTTTTAAATAATTTTCAATAATGTCGCCAATATCGTAGTCATCTT

**AdeA19606** TGCAATTCAATCGCTTGCTTTCCATTCATGGCCCGAATAACACTCATGCCTTCACGTTTTAAATAATTTTCAATAATGTCGCCAATATCGTAGTCATCTT

**AdeA17961** TGCAATTCAATCGCTTGCTTTCCATTCATGGCCCGAATAACACTCATGCCTTCACGTTTTAAATAATTTTCAATAATGTCGCCAATATCGTAGTCATCTT

***310 320 330 340 350 360 370 380 390 400***

***....|....|....|....|....|....|....|....|....|....|....|....|....|....|....|....|....|....|....|....|***

**AdeA17978** CTACCACAAGAATAACTTTATCTTGGCAATCAAAAGAAAAAGAATGATCAAACATAGAAAATCTGGCTATAGAAAGTGCTTCAACTCATCATACGCTAAA

**AdeA19606** CTACCACAAGAATAACTTTATCTTGGCAATCAAAAGAAAAAGAATGATCAAACATAGATAATCTGGCTATAGAAAGTGCTTCAACTCATCATACGCTAAA

**AdeA17961** CTACCACAAGAATAACTTTATCTTGGCAATCAAAAGAAAAAGAATGATCAAACATAGATAATCTGGCTATAGAAAGTGCTTCAACTCATCATACGCTAAA

***410 420 430 440 450 460 470 480 490 500***

***....|....|....|....|....|....|....|....|....|....|....|....|....|....|....|....|....|....|....|....|***

**AdeA17978** TTATCCGTATTTCTCCACACTTACTCCACACTTTAGTGATTATCCCTACACACTCATCAAAAATAATACGAACATCAAAAACTCACTAGGTTTGGACAGT

**AdeA19606** TTATCCGTATTTCTCCACACTTACTCCACACTTTAGTGATTATCCCTACACACTCATCAAAAATAATACGAACATCAAAAACTCACTAGGTTTGGACAGT

**AdeA17961** TTATCCGTATTTCTCCACACTTACTCCACACTTTAGTGATTATCCCTACACACTCATCAAAAATAATACGAACATCAAAAACTCACTAGGTTTGGACAGT

***...***

**AdeA17978** ATG

**AdeA19606** ATG

**AdeA17961** ATG

**Supplementary Figure 1**. Alignments of *adeABC* promoter sequences. Conserved sequence is shown in grey. TATA box (yellow), RBS (green), AdeR binding operator repeat (pink), and translational start site (cyan) are indicated according to (1, 2).

***10 20 30 40 50 60 70 80 90 100***

***....|....|....|....|....|....|....|....|....|....|....|....|....|....|....|....|....|....|....|....|***

**AdeF17978** CGATACAGGCACATCAATACGAAGCTGACCTCTTGGCCCCCGCTCTGCATCATGAAAAGAAGATTCAATATCGGCAACgTCCGCTAAAATACGGGCTGTC

**AdeF19606** CGATACAGGCACATCAATACGAAGCTGACCTCTTGGCCCCCGCTCTGCATCATGAAAAGAAGATTCAATATCGGCAACATCCGCTAAAATACGGGCTGTC

**AdeF17961** CGATACAGGCACATCAATACGAAGCTGACCTCTTGGCCCCCGCTCTGCATCATGAAAAGAAGATTCAATATCGGCAACATCCGCTAAAATACGGGCTGTC

***110 120 130 140 150 160 170 180 190 200***

***....|....|....|....|....|....|....|....|....|....|....|....|....|....|....|....|....|....|....|....|***

**AdeF17978** CGATCATAATATACGGCGCCATCCGGTGTGAGACTAATTTTTCGTGTTGTCCGATTAAGCAATCGAACTTGTAAATGCTTCTCTAAAGCCTGAATGGTTG

**AdeF19606** CGATCATAATATACGGCGCCATCCGGTGTGAGACTAATTTTTCGTGTTGTCCGATTAAGCAATCGAACTTGTAAATGCTTCTCTAAAGCCTGAATGGTTG

**AdeF17961** CGATCATAATATACGGCGCCATCCGGTGTGAGACTAATTTTTCGTGTTGTCCGATTAAGCAATCGAACTTGTAAATGCTTCTCTAAAGCCTGAATGGTTG

***210 220 230 240 250 260 270 280 290 300***

***....|....|....|....|....|....|....|....|....|....|....|....|....|....|....|....|....|....|....|....|***

**AdeF17978** TAGTCACAGAAGCACGCGGTAAACtCAAACTTCAGCTGCTAAACTGAAACTATTTGTTTCAACAACTTTGTTGAATACTCTCATGGCATGAAATAGATCC

**AdeF19606** TAGTCACAGAAGCACGCGGTAAACCCAAACTTCAGCTGCTAAACTGAgACTATTTGTTTCAACAACTTTGTTGAATACTCTCATGGCATGAAATAGATCC

**AdeF17961** TAGTCACAGAAGCACGCGGTAAACCCAAACTTCAGCTGCTAAACTGAAACTATTTGTTTCAACAACTTTGTTGAATACTCTCATGGCATGAAATAGATCC

***310 320 330 340 350 360 370 380 390 400***

***....|....|....|....|....|....|....|....|....|....|....|....|....|....|....|....|....|....|....|....|***

**AdeF17978** ACACGCACACTCCTATTAATGCATTTTCTAGTGCAAACACATCAATTGTTATAAAAATgCGATAGTGTTATCAAgTTTAATATATTTATTCGTGATTGAC

**AdeF19606** ACACGCACACTCCTATTAATGCATTTTCTAGTGCAAACACATCAATTGTTATAAAAATACGATAGTGTTATCAAATTTAATATATTTATTCGTGATTGAC

**AdeF17961** ACACGCACACTCCTATTAATGCATTTTCTAGTGCAAACACATCAATTGTTATAAAAATACGATAGTGTTATCAAATTTAATATATTTATTCGTGATTGAC

***410 420 430 440 450 460 470 480 490***

***....|....|....|....|....|....|....|....|....|....|....|....|....|....|....|....|....|....|....|..***

**AdeF17978** AAACTTTTATGCTCCGTCCATTCCATAAACCCTGTTTCTCCCATACGGACCATAACTAAAAGTCTGAAAACAGGTATCCAAATAACTAGGAGCACCT

**AdeF19606** AAACTTTTATGCTCCGTCCATTCCATAAACCCTGTTTCTCCCATACGGACCATAACTAAAAGTCTGAAAACAGGTATCCAAATAACTAGGAGCACCT

**AdeF17961** AAACTTTTATGCTCCGTCCATTCCATAAACCCTGTTTCTCCCATACGGACCATAACTAAAAGTCTGAAAACAGGTATCCAAATAACTAGGAGCACCT

**Supplementary Figure 2**. Alignments of *adeFGH* promoter sequences. Conserved sequence is shown in grey. TATA box (yellow), and RBS (green) are indicated according to (3).

***10 20 30 40 50 60 70 80 90 100***

***....|....|....|....|....|....|....|....|....|....|....|....|....|....|....|....|....|....|....|....|***

**AdeI17978** TTGCACGCGTAGGCGGCATGCCTTTTGTATGTTTTTTATCCTTTCTAGTATGTATATATTTAGCATGGcATAAAAAATATATTACTGTTATTTTCATTAG

**AdeI19606** TTGCACGCGTAGGCGGCATGCCTTTTGTATGTTTTTTATCCTTTCTAGTATGTATATATTTAGCATGGTATAAAAAATATATTACTGTTATTTTCATTAG

**AdeI17961** TTGCACGCGTAGGCGGCATGCCTTTTGTATGTTTTTTATCCTTTCTAGTATGTATATATTTAGCATGGTATAAAAAATATATTACTGTTATTTTCATTAG

***110 120 130 140 150 160 170 180 190 200***

***....|....|....|....|....|....|....|....|....|....|....|....|....|....|....|....|....|....|....|....|***

**AdeI17978** CTTGGGGGTTATTGGCAGTATCACCATGGGTTGGCTGCTCAAGTGGTGTGTTAACCGGCCTAGACCTCCTGAGGCATATCATATTGTTGcAAGTTACGGT

**AdeI19606** CTTGGGAGTTATTGGCAGTATCACCATGGGTTGGCTGCTCAAGTGGTGTGTTAACCGGCCTAGACCTCCTGAGGCATATCATATTGTTGAAAGTTACGGT

**AdeI17961** CTTGGGAGTTATTGGCAGTATCACCATGGGTTGGCTGCTCAAGTGGTGTGTTAACCGGCCTAGACCTCCTGAGGCATATCATATTGTTGAAAGTTACGGT

***210 220 230 240 250 260 270 280 290 300***

***....|....|....|....|....|....|....|....|....|....|....|....|....|....|....|....|....|....|....|....|***

**AdeI17978** GCATCGTTCCCAAGTGCACATAGTGTTTATGCATCAACACTGGCTTGTCTGGCAATGATTATGTTATGCCATAAGCtCAACATTAACTCTCCTTATATTG

**AdeI19606** GCATCGTTCCCAAGTGCACATAGTGTTTATGCATCAACACTGGCTTGTCTGGCAATGATTATGTTATGCCATAAGCACAACATTAACTCTCCTTATATTG

**AdeI17961** GCATCGTTCCCAAGTGCACATAGTGTTTATGCATCAACACTGGCTTGTCTGGCAATGATTGTGTTATGCCATAAGCACAACATTAACTCTCCTTATATTG

***310 320 330 340 350 360 370 380 390 400***

***....|....|....|....|....|....|....|....|....|....|....|....|....|....|....|....|....|....|....|....|***

**AdeI17978** TTTTGATCTCCTGTCTTTGGTTTGTGTGTATGGGGCTTTCAAGAATATATGCAGGAGTTCATTTCCCAACAGACGTACTCGCTGGTTGGGGCATTGGTTT

**AdeI19606** TTTTGATCTCCTGTCTTTGGTTTGTGTGTATGGGGCTTTCAAGAATATATGCAGGAGTTCATTTCCCAACAGACGTACTCGCTGGTTGGGGCATTGGTTT

**AdeI17961** TTTTGATCTCCTGTCTTTGGTTTGTGTGTATGGGGCTTTCAAGAATATATGCAGGAGTTCATTTCCCAACAGACGTACTCGCTGGTTGGGGCATTGGTTT

***410 420 430 440 450 460 470 480 490***

***....|....|....|....|....|....|....|....|....|....|....|....|....|....|....|....|....|....|....|....***

**AdeI17978** TATTTaGATTGCACTGCTTTGGCTCTGGTTATTACAAACACAAAGTAGGTTAAGTAGAAAACAAATATATTTTTAGATTTTATCTAAACGAGGTGGAAC

**AdeI19606** TATTTGGATTGCACTGCTTTGGCTCTGGTTATTACAAACACAAAGTAGGTTAAGTAGAAAACAAATATATTTTTAGATTTTATCTAAACGAGGTGGAAC

**AdeI17961** TATTTGGATTGCACTGCTTTGGCTCTGGTTATTACAAACACAAAGTAGGTTAAGTAGAAAACAAATATATTTTTAGATTTTATCTAAACGAGGTGGAAC

**Supplementary Figure 3**. Alignments of *adeIJK* promoter sequences. Conserved sequence is shown in grey. TATA box (yellow), and RBS (green) are indicated according to (4).

***10 20 30 40 50 60 70 80 90 100***

***....|....|....|....|....|....|....|....|....|....|....|....|....|....|....|....|....|....|....|....|***

**AdeR17978** CTTTGAGTCTTGCtACCTCAGCTTCAGCTTTATTGAGAGAAGCTCTATTGCTATTTACATCGGCCTCAAAAGTCTCGGAATTAATTTTATATAAGGCTTG

**AdeR19606** CTTTGAGTCTTGCTACCTCAGCTTCAGCTTTATTGAGAGAAGCTCTATTGCTATTTACATCTGCCTCAAAAGTCTCGGAATTAATTTTATATAAAGCTTG

**AdeR17961** CTTTGAGTCTTGCTACCTCAGCTTCAGCTTTATTGAGAGAAGCTCTATTGCTATTTACATCTGCCTCAAAAGTCTCGGAATTAATTTTATATAAAGCTTG

***110 120 130 140 150 160 170 180 190 200***

***....|....|....|....|....|....|....|....|....|....|....|....|....|....|....|....|....|....|....|....|***

**AdeR17978** CCCTGCTCTAACTTCACTACCTTGTTTAAATAGAACCTTTTCAATGATACCTCCGACTTGCGGACGGATTTCCGCCGTACGGAACGCATGTACTCGTGCA

**AdeR19606** TCCTGCTCTAACTTCACTACCCTGTTTAAATAAAACCTTTTCAATGATACCTCCGACTTGCGGACGGATTTCCGCCGTACGGAATGCATGTACACGTGCA

**AdeR17961** TCCTGCTCTAACTTCACTACCCTGTTTAAATAAAACCTTTTCAATGATACCTCCGACTTGCGGACGGATTTCCGCCGTACGGAATGCATGTACACGTGCA

***210 220 230 240 250 260 270 280 290 300***

***....|....|....|....|....|....|....|....|....|....|....|....|....|....|....|....|....|....|....|....|***

**AdeR17978** GGAAGATTTTCACTAAAATTTACCGATTGCGGTTGAATGCTTAATACACTGACTTTAGCCGGTGGTGGCTCAGCTTGAGCGACTTTTTTTGAATCACACC

**AdeR19606** GGAAGATTTTCACTAAAATTTACCGATTGCGGTTGAATGCTTAATACACTGACTTTAGCCGGTGGTGGCTCAGCTTGAGCGACTTCTTTTGAATCACACC

**AdeR17961** GGAAGATTTTCACTAAAATTTACCGATTGCGGTTGAATGCTTAATACACTGACTTTAGCCGGTGGTGGCTCAGCTTGAGCGACTTCTTTTGAATCACACC

***310 320 330 340 350 360 370 380 390 400***

***....|....|....|....|....|....|....|....|....|....|....|....|....|....|....|....|....|....|....|....|***

**AdeR17978** CCTGTAATATCAGCCCAATAGATAAAAATAAAGGAAGTAAAAGATGCTTTTGCATACTGTCCAAACCTAGTGAGTTTTTGATGTTCGTATTATTTTTGAT

**AdeR19606** CCTGTAATATCAGCCCAATAGATAAAAATAAAGGAAGTAAAAGATGCTTTTGCATACTGTCCAAACCTAGTGAGTTTTTGATGTTCGTATTATTTTTGAT

**AdeR17961** CCTGTAATATCAGCCCAATAGATAAAAATAAAGGAAGTAAAAGATGCTTTTGCATACTGTCCAAACCTAGTGAGTTTTTGATGTTCGTATTATTTTTGAT

***410 420 430 440 450 460 470 480 490***

***....|....|....|....|....|....|....|....|....|....|....|....|....|....|....|....|....|....|....|....***

**AdeR17978** GAGTGcGTAGGGATAATCACTAAAGTGTGGAGTAAGTGTGGAGAAATACGGATAATTTAGCGTATGATGAGTTGAAGCACTTTCTATAGCCAGATTaTC

**AdeR19606** GAGTGTGTAGGGATAATCACTAAAGTGTGGAGTAAGTGTGGAGAAATACGGATAATTTAGCGTATGATGAGTTGAAGCACTTTCTATAGCCAGATTATC

**AdeR17961** GAGTGTGTAGGGATAATCACTAAAGTGCGGAGTAAGTGTGGAGAAATACGGATAATTTAGCGTATGATGAGTTGAAGCACTTTCTATAGCCAGATTATC

**Supplementary Figure 4**. Alignments of *adeRS* promoter sequences.

**References**

1. Chang T-Y, Huang B-J, Sun J-R, et al. AdeR protein regulates adeABC expression by binding to a direct-repeat motif in the intercistronic spacer. Microbiol Res 2016; 183: 60–7. (PMID: 26805619)

2. Marchand I, Damier-Piolle L, Courvalin P, Lambert T. Expression of the RND-type efflux pump AdeABC in *Acinetobacter baumannii* is regulated by the AdeRS two-component system. Antimicrob Agents Chemother 2004; 48: 3298–304. (PMID: 15328088)

3. Coyne S, Guigon G, Courvalin P, Perichon B. Screening and quantification of the expression of antibiotic resistance genes in *Acinetobacter baumannii* with a microarray. Antimicrob Agents Chemother 2010; 54: 333–40. (PMID: 19884373)

4. Kröger C, MacKenzie KD, Alshabib EY, et al. The primary transcriptome, small RNAs and regulation of antimicrobial resistance in *Acinetobacter baumannii* ATCC 17978. Nucleic Acids Res 2018; 46: 9684–98. (PMID: 29986115)
